# Supplementary material for: Soil carbon in the world’s tidal marshes
Source: Nat Commun. 2024 Nov 26;15:10265. doi: 10.1038/s41467-024-54572-9 (PMC11599748; doi:10.1038/s41467-024-54572-9)
Supplement: Supplementary file 1 — Supplementary Information [file 41467_2024_54572_MOESM1_ESM.pdf]

Supplementary Information: Soil carbon in the world's tidal marshes

Tania L. Maxwell[1,31,*], Mark D. Spalding[1,2], Daniel A. Friess[3], Nicholas J. Murray[4], Kerrylee Rogers[5], Andre S. Rovai[6,7], Lindsey S. Smart[8,9], Lukas Weilguny[10], Maria Fernanda Adame[11], Janine B. Adams[12,13], William E.N. Austin[14,15], Margareth S. Copertino[16,17], Grace M. Cott[18], Micheli Duarte de Paula Costa[19], James R. Holmquist[20], Cai J.T. Ladd[21,22], Catherine E. Lovelock[23], Marvin Ludwig[24], Monica M. Moritsch[25], Alejandro Navarro[4], Jacqueline L. Raw[13,26], Ana-Carolina Ruiz-Fernández[27], Oscar Serrano[28], Craig Smeaton[14], Marijn Van de Broek[29], Lisamarie Windham-Myers[30], Emily Landis[8], and Thomas A. Worthington[1,*]

[1] Conservation Science Group, Department of Zoology, University of Cambridge, Cambridge, UK.
[2] The Nature Conservancy, Siena, Italy.
[3] Department of Earth and Environmental Sciences, Tulane University, New Orleans, LA, USA.
[4] College of Science and Engineering, James Cook University, Townsville, QLD, Australia.
[5] Environmental Futures, School of Science, University of Wollongong, Wollongong, NSW, Australia.
[6] U.S. Army Engineer Research and Development Center, Vicksburg, MS, USA.
[7] Department of Oceanography and Coastal Sciences, Louisiana State University, Baton Rouge, LA, USA.
[8] The Nature Conservancy, Arlington, VA, USA.
[9] Department of Forestry and Environmental Resources, NC State University, Raleigh, NC, USA.
[10] European Molecular Biology Laboratory, European Bioinformatics Institute, Wellcome Genome Campus, Hinxton, UK.
[11] Australian Rivers Institute, Coastal and Marine Research Centre, Griffith University, Nathan, QLD, Australia.
[12] Department of Botany, Nelson Mandela University, Gqeberha, South Africa.
[13] Institute for Coastal and Marine Research, Nelson Mandela University, Gqeberha, South Africa.
[14] School of Geography and Sustainable Development, University of St Andrews, St Andrews, UK.
[15] Scottish Association of Marine Science, Oban, UK.
[16] Federal University of Rio Grande (FURG), Rio Grande, Brazil.
[17] Brazilian Network of Climate Change Studies—Rede CLIMA, Rio Grande, Brazil.
[18] School of Biology and Environmental Science, University College Dublin, Belfield, Ireland.
[19] Deakin Marine Research and Innovation Centre, School of Life and Environmental Sciences, Deakin University, Warrnambool, VIC, Australia.
[20] Smithsonian Environmental Research Center, Edgewater, MD, USA.
[21] Department of Geography, Swansea University, Swansea, UK.
[22] School of Ocean Sciences, Bangor University, Menai Bridge, UK.
[23] School of Environment, The University of Queensland, St Lucia, QLD, Australia.
[24] Institute of Landscape Ecology, University of Münster, Münster, Germany.
[25] University of California, Santa Cruz, Santa Cruz, CA, USA.
[26] Anthesis South Africa, Cape Town, South Africa.

[27] Instituto de Ciencias del Mar y Limnología, Universidad Nacional Autónoma de México, Unidad Académica Mazatlán, Mazatlán, Mexico.
[28] Centro de Estudios Avanzados de Blanes (CEAB), Blanes, Spain.
[29] Swiss Federal Institute of Technology (ETH Zürich), Zürich, Switzerland.
[30] California Delta Stewardship Council, Sacramento, CA, USA.
[31] Present address: Biodiversity, Ecology and Conservation Research Group, International Institute for Applied Systems Analysis (IIASA), Laxenburg, Austria.
* taniamaxwell7@gmail.com, taw52@cam.ac.uk

Figures

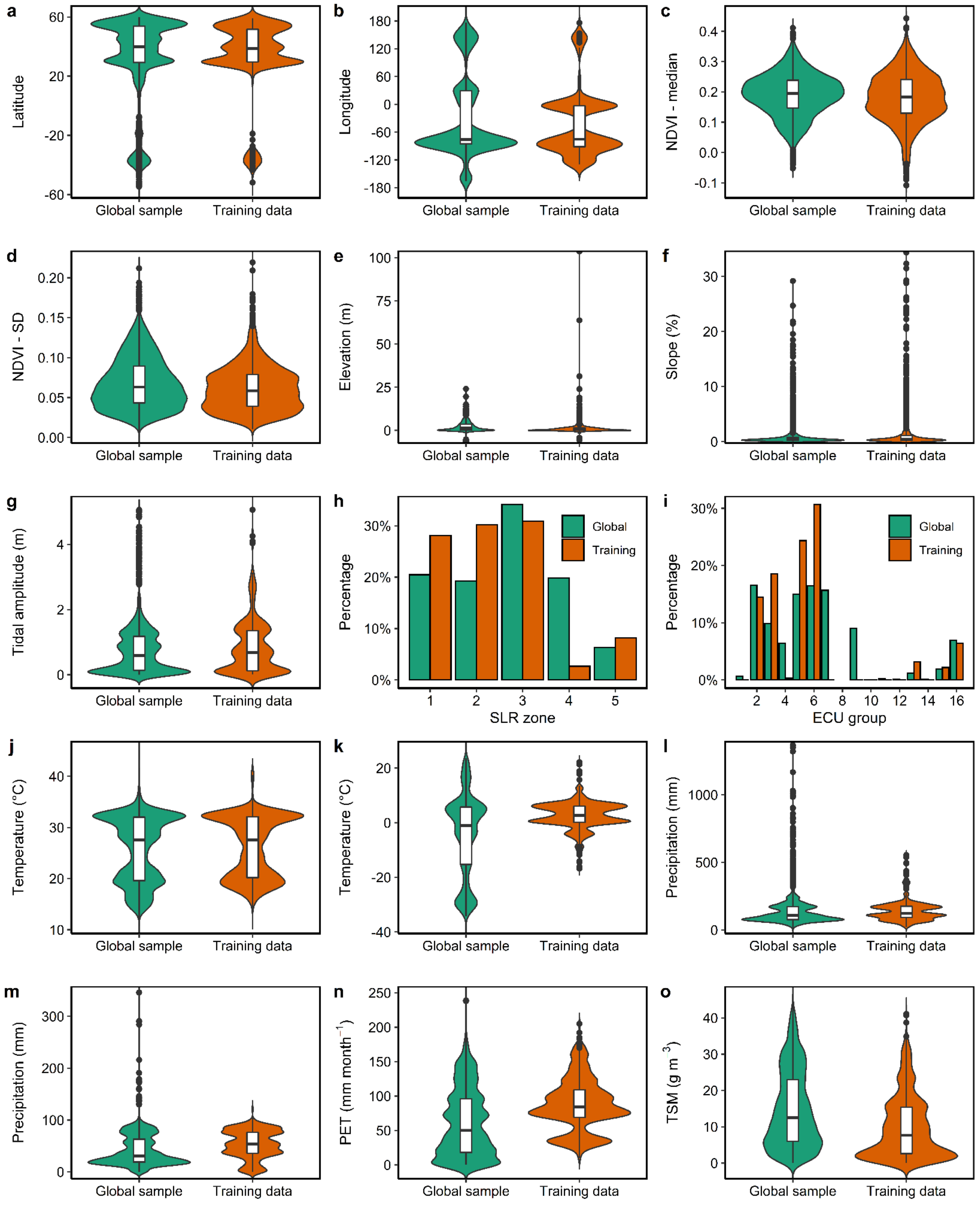

Fig. S1 | The distribution of values for the environmental covariates of the training data (orange) in comparison to 10,000 random points sampled across the global tidal marsh extent (green). a) Latitude, b) Longitude, c) Median of Normalised Difference Vegetation Index (NDVI), d) Standard deviation (SD) of Normalised Difference Vegetation Index (NDVI), e) Elevation, f) Slope, g) Tidal amplitude, h) Sea-level rise (SLR) zone, i) Coastal morphology group, j) Maximum temperature of the warmest month, k) Minimum temperature of the coldest month l) Precipitation of the wettest month m) Precipitation of the driest month n) Potential

evapotranspiration (PET) of the driest quarter and o) Total suspended matter (TSM). Violin plots display the probability density of the data at different values along the y-axis, with the overlaying boxplots showing median value (horizontal line), box ends representing the upper and lower quartiles and thin lines highest and lowest values excluding outliers (outside 1.5 times the interquartile range above the upper quartile and below the lower quartile), outliers shown as black dots. Bar plots represent the percentage of data in different groups of SLR zone and coastal morphology ecological coastal units (ECUs).

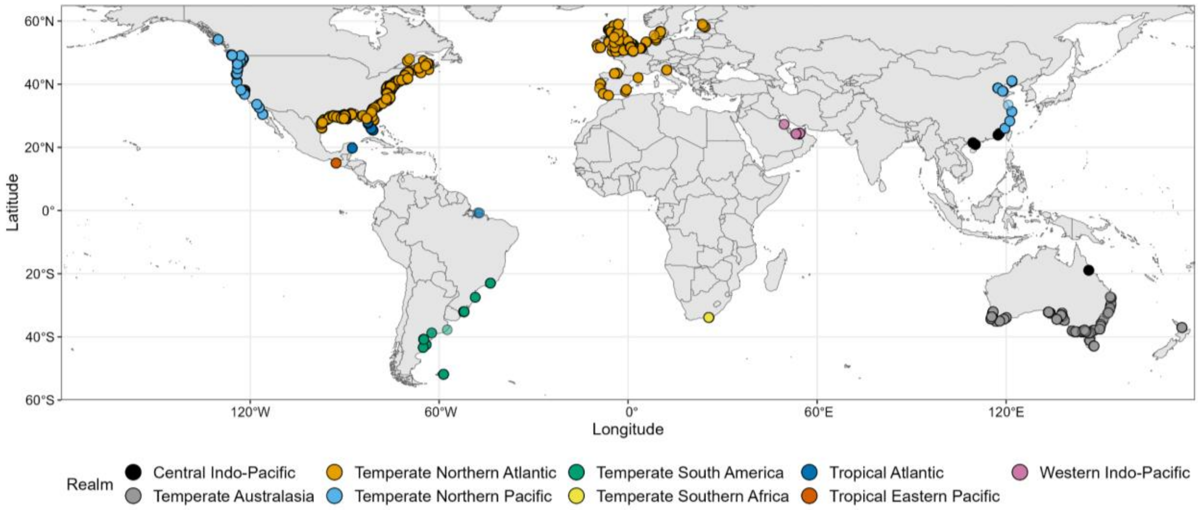

Fig. S2 | Locations of training data points. Training data categorized by biogeographical realms of the Marine Ecoregions of the World[1]. The arctic and eastern Indo-Pacific realms are not represented due to lack of data in these regions.

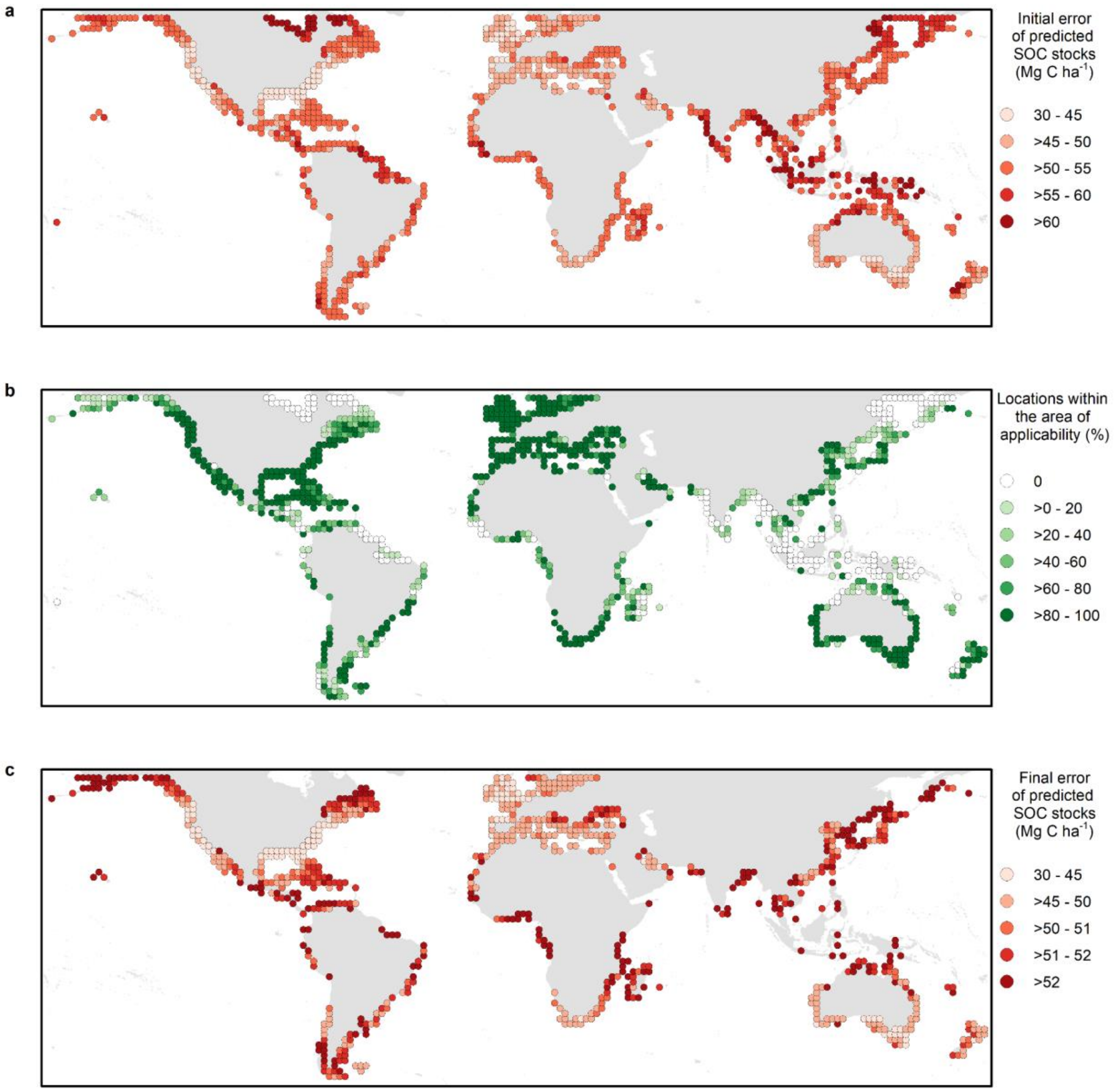

Fig. S3 | Global distribution of expected error of the tidal marsh soil organic carbon (SOC) predictions for the 0-30 cm soil layer (aggregated to 2°). a) Initial expected model error of predicted SOC per unit area (megagrams carbon per hectare (Mg C ha^{-1})), without taking into account whether predictions were meaningful. b) The proportion of pixels located within the area of applicability (AOA), i.e. where we enabled the model to learn about the relationship between SOC and the environmental drivers for this 0-30 cm soil layer. c) Final expected model error of predicted SOC per unit area (megagrams carbon per hectare (Mg C ha^{-1})), after masking out pixels outside the AOA. NB. Please note the difference in error classes between panels a) and c).

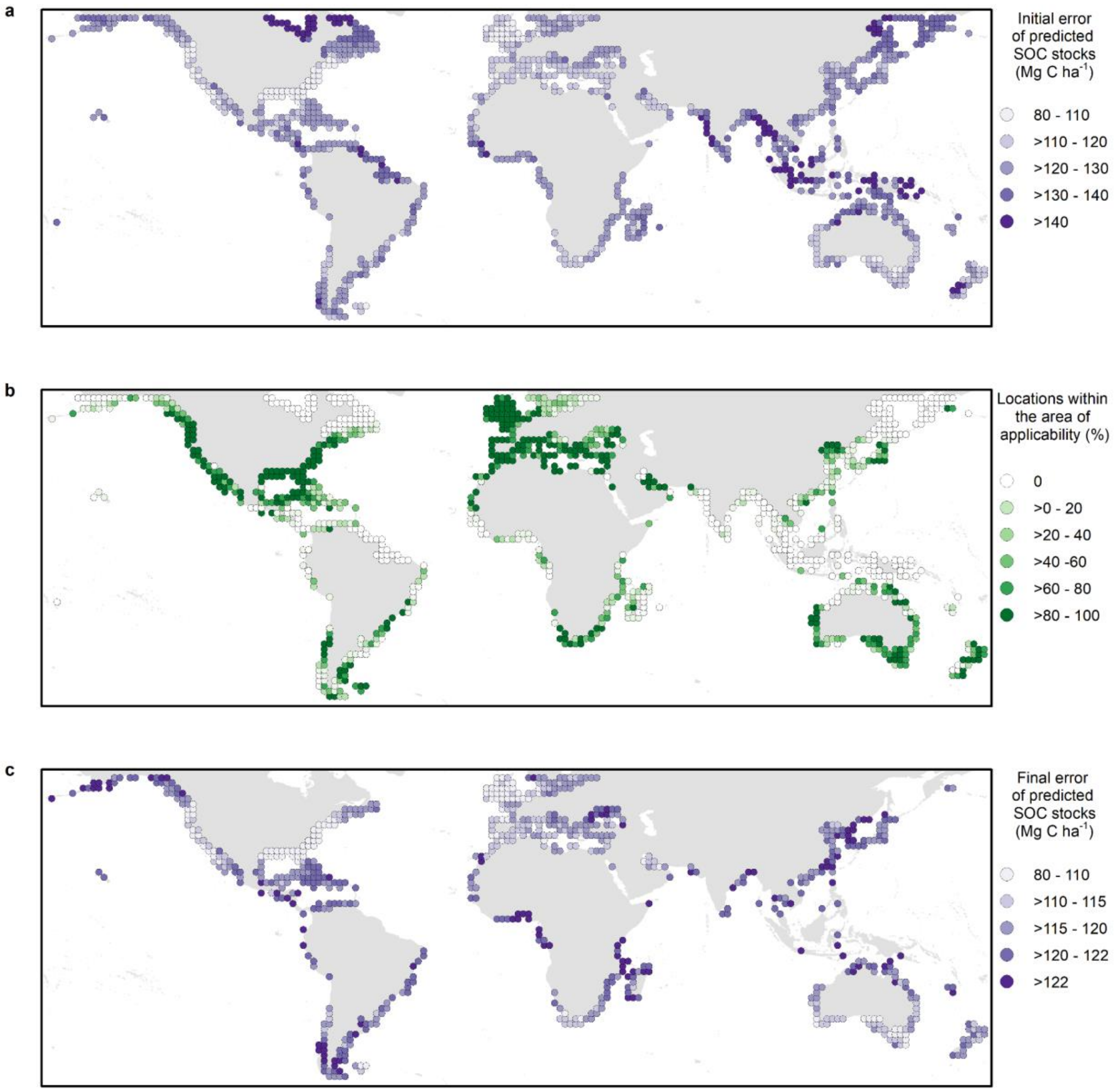

Fig. S4 | Global distribution of expected error of the tidal marsh soil organic carbon (SOC) predictions for the 30-100 cm soil layer (summarised to 2°). a) Initial expected model error of predicted SOC per unit area (megagrams carbon per hectare (Mg C ha^{-1})), without taking into account whether predictions were meaningful. b) The proportion of pixels located within the area of applicability (AOA), i.e. where we enabled the model to learn about the relationship between SOC and the environmental drivers for this 0-30 cm soil layer. c) Final expected model error of predicted SOC per unit area (megagrams carbon per hectare (Mg C ha^{-1})), after masking out pixels outside the AOA. NB. Please note the difference in error classes between panels a) and c).

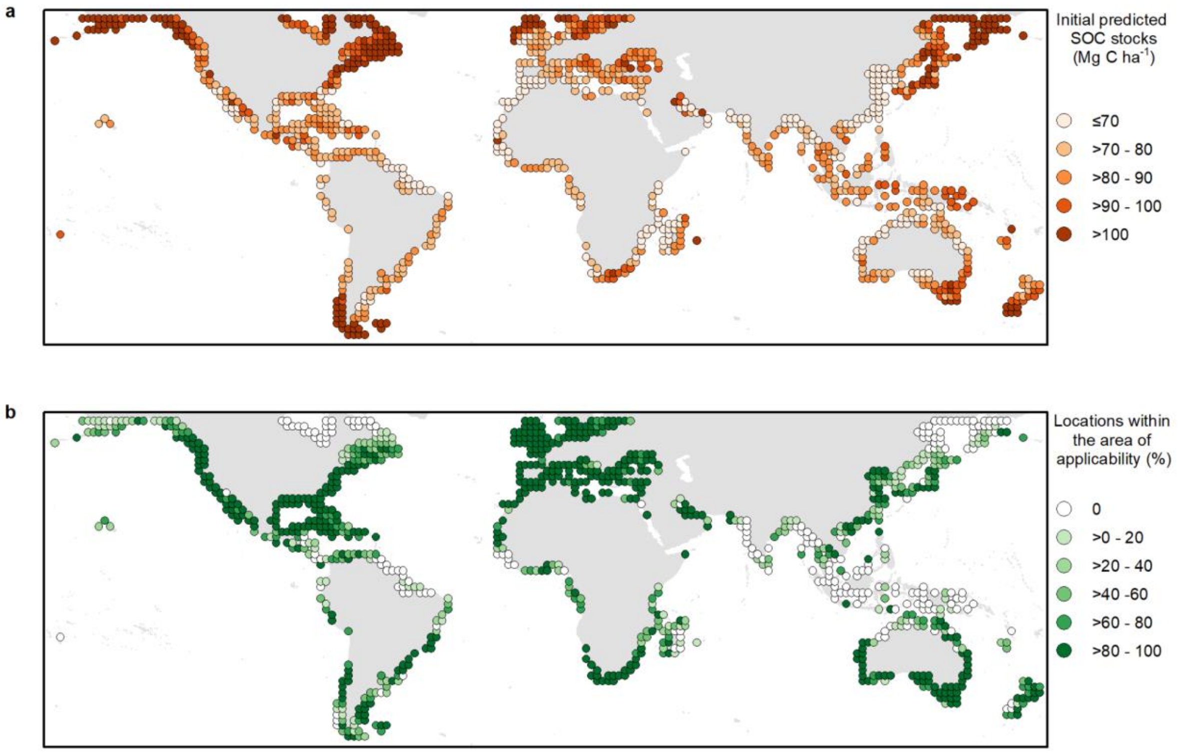

Fig. S5 | Global distribution of tidal marsh soil organic carbon (SOC) for the 0-30 cm soil layer (aggregated per 2° cell). a) Initial predicted SOC per unit area (megagrams carbon per hectare (Mg C ha^{-1})). b) The proportion of pixels located within the area of applicability (AOA), i.e. where we enabled the model to learn about the relationship between SOC and the environmental drivers for this 0-30 cm soil layer. The final predicted SOC per unit area (megagrams carbon per hectare (Mg C ha^{-1})), after removing pixels outside the AOA, are presented in Fig. 2a.

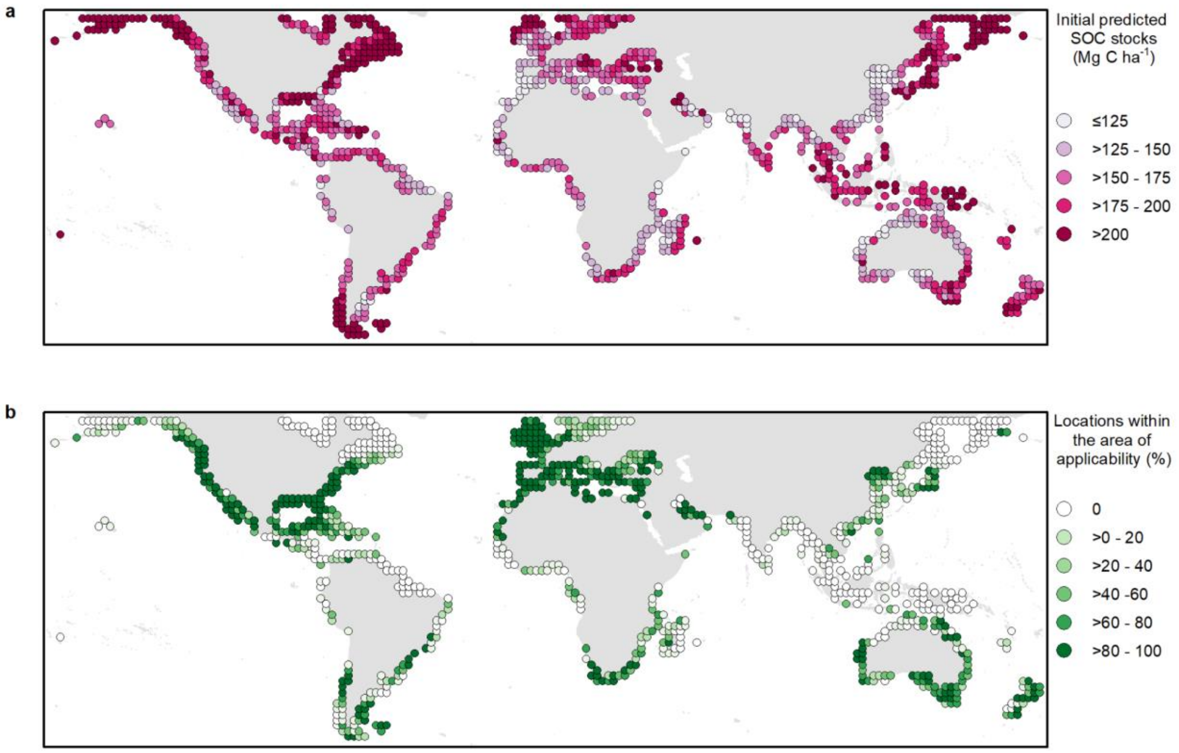

Fig. S6 | Global distribution of tidal marsh soil organic carbon (SOC) for the 30-100 cm soil layer (summarised per 2° cell). a) Initial predicted SOC per unit area (megagrams carbon per hectare (Mg C ha^{-1})). b) The proportion of pixels located within the area of applicability (AOA), i.e. where we enabled the model to learn about the relationship between SOC and the environmental drivers for this 30-100 cm soil layer. The final predicted SOC per unit area (megagrams carbon per hectare (Mg C ha^{-1})), after removing pixels outside the AOA, are presented in Fig. 2b.

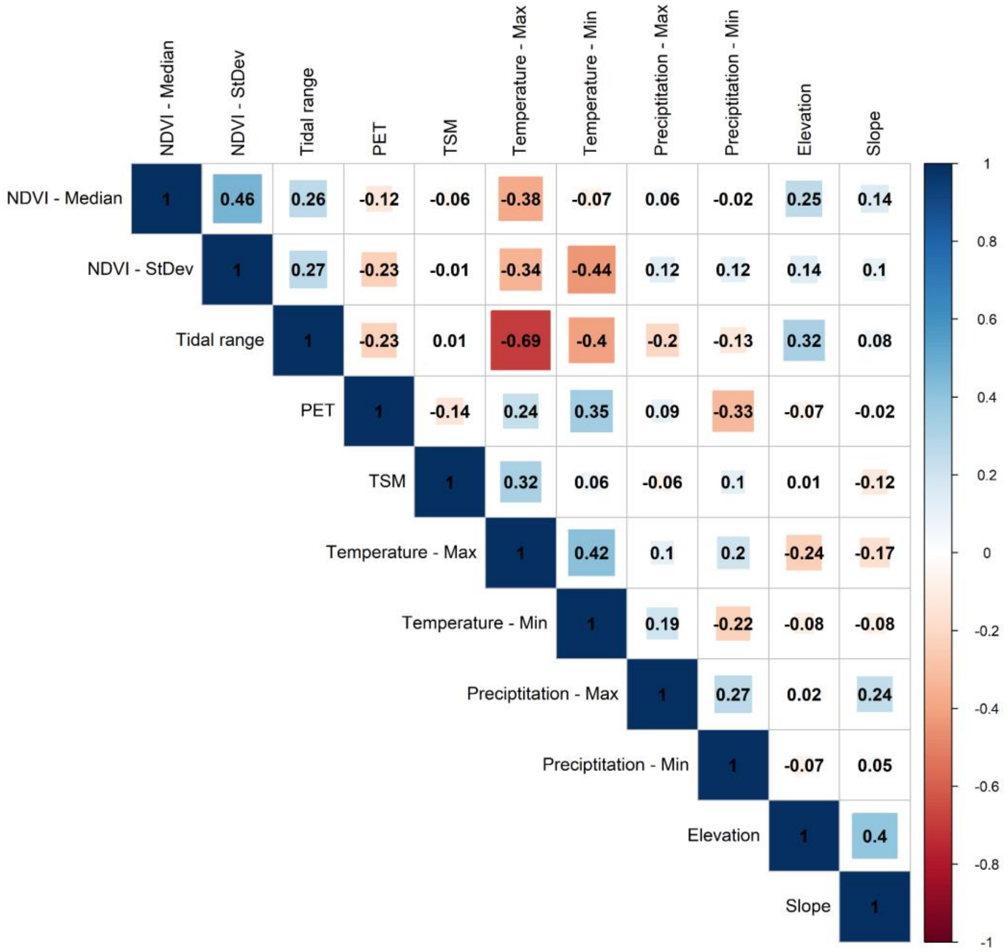

Fig. S7 | Correlation between continuous variables used in the random forest model. Colours represent the strength and direction of the correlation, correlation coefficient given for each variable pair.

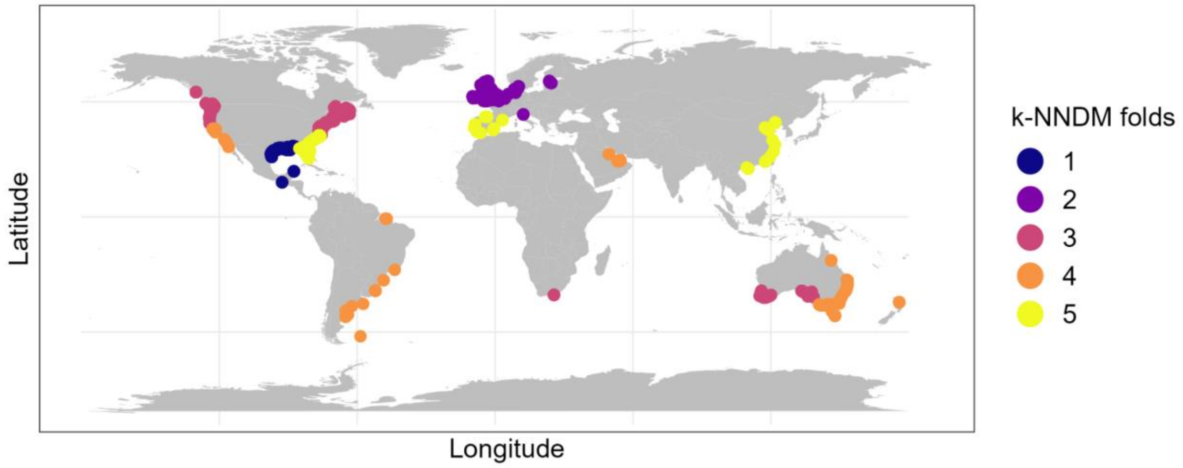

Fig. S8 | Training data divided into 5 folds for the k-fold Nearest Neighbour Distance Matching (k-NNDM) Cross-Validation. Approach follows methods described by Linnenbrink et al.[2].

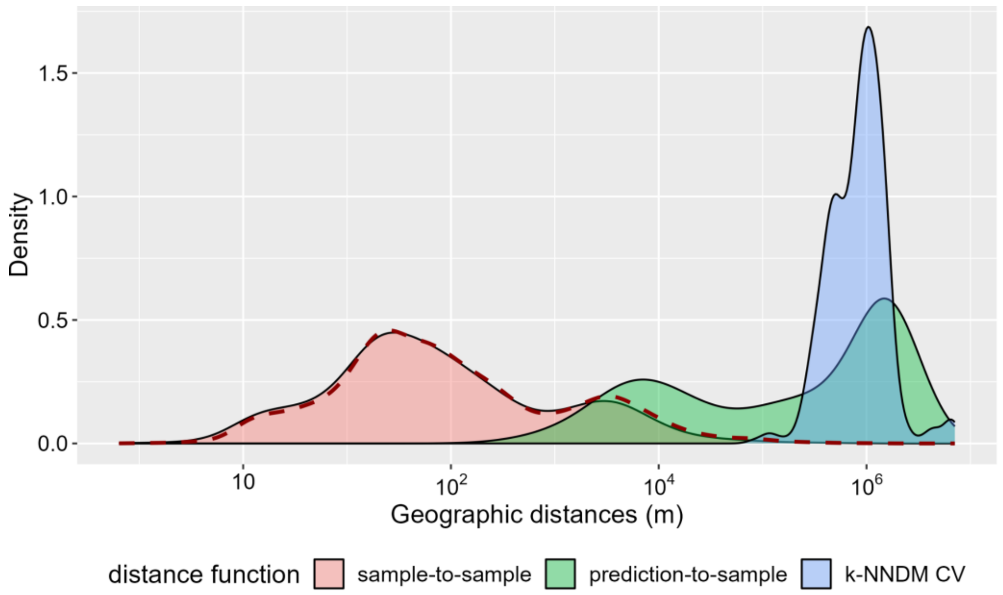

Fig. S9 | Comparison of the geographic distance between folds of the random cross validation (dashed red line). This reproduces the distances between the samples (pink), and the k-fold nearest neighbour distance matching (k-NNDM) cross validation (blue), which better resembles the distance from prediction locations to training samples (green).

Tables

Table S1 | Hypothesized landscape-level drivers of soil organic carbon (SOC) in tidal marshes globally. These variables were selected using expert opinion and discussion, along with previous studies investigating the variables identified for their associations with SOC in vegetated coastal ecosystems[3–5], and supported by evidence from the published literature.

**Landscape-level drivers**	**Relation to SOC in tidal marshes**	**Spatial data description**	**Number of Variables**	**Spatial data source**
ECOLOGICAL				
Vegetation class	C stocks vary across species[6]. Species with rhizomes (graminoids) have higher SOC density. Indices such as NDVI are capable of discriminating between broad tidal marsh vegetation communities.	Use NDVI as a proxy for source of SOC. Assessed as median and standard deviation. Calculated from Landsat 8 bands from 2014 to 2021.<br><br>→ resolution 30 m	2	ee.ImageCollection("LANDSAT/LC08/C02/T1_L2")<br><br>Pre-processed using code from Murray et al. (2022)[7,8]
GEOMORPHOLOGICAL				
Elevation (m)	Higher SOC stocks are generally found in lower elevation due (1) higher sedimentation rates allowing more trapping of organic C from surface organisms[9] and (2) more frequent inundation providing opportunity to settle more allochthonous C particles[10]. However, sea level rise history may lead to higher C stocks in the intertidal marshes[11,12].	Copernicus DEM<br>→ resolution 30 m	1	ee.ImageCollection("COPERNICUS/DEM/GLO30")<br>https://spacedata.copernicus.eu/documents/20123/121239/GEO1988-CopernicusDEM-SPE-002_ProductHandbook_I4.0.pdf
Slope (%)	Higher SOC stocks are associated with shallower slopes, due to lower risk of erosion compared to steeper slopes[13].	Calculated from elevation data	1	
Tidal amplitude (m)	Can influence the stability and resilience of marshes[13], as well as accommodation space[14].	FES2014 Tide Model M2<br>→ resolution 1/16 degree (~7 km)	1	https://datastore.cls.fr/catalogues/fes2014-tide-model/
Sea-level rise	Higher SOC stocks further from estuaries can be explained by the signature of past sea level rise[11].	Holocene relative sea-level rise zones<br>→ 5 broad-scale zones	1	Clark et al.[15]
Coastal morphology	Each coastal setting (deltas, estuaries, lagoons, composite deltas and lagoons, bedrock, and carbonate) has an apparent environmental signature that controls the SOC stock *via* the type and rate of sediment supply to the coastline, nutrient loading/limitation, and organic matter diagenesis[16].	Ecological coastal units<br>→ 16 classes	1	https://www.esri.com/arcgis-blog/products/arcgis-living-atlas/mapping/ecus-available/
OTHER ENVIRONMENTAL				

Temperature	Higher temperatures generally increase the productivity and growth of vegetation[17], and are associated with higher SOC stocks[18].<br><br>Minimum temperature and minimum precipitation were chosen rather than mean annual values[16], as they portray environmental thresholds that may have a stronger effect on SOC stocks by constraining ecosystem functionality[19], which regulates both production and decomposition rates.	Assessed as minimum temperature of coldest month and maximum temperature of warmest month (°C).<br><br>WorldClim BIO5 & BIO6<br>→ resolution 927.67 meters	2	https://www.worldclim.org/data/bioclim.html<br><br>ee.Image("WORLDCLIM/V1/BIO")
Precipitation	Higher rainfall is generally associated with higher SOC by increasing the freshwater runoff and thus potentially higher deposition of allochthonous organic matter[13].	Assessed as precipitation of driest month (mm) and precipitation of wettest month (mm).<br><br>WorldClim BIO13 & BIO14<br>→ resolution 927.67 meters	2	https://www.worldclim.org/data/bioclim.html<br><br>ee.Image("WORLDCLIM/V1/BIO")
Potential evapotranspiration (PET)	Potential evapotranspiration has been found to explain ecophysiological processes in mangroves, a neighbouring coastal ecosystem. The potential evapotranspiration can influence the concentration of salts and nutrients in the soils and groundwater in wetlands[20].	Assessed as PET of the driest quarter.<br><br>ENVIREM<br>→ resolution 927.67 meters	1	https://envirem.github.io/#download
Total suspended matter	More sediment is associated with lower C density but deeper deposits, along with higher accretion and C sequestration[21].	Derived from remotely sensed MERIS, reprocessed from GlobColour project<br>→ Resolution 4 km<br>→ Monthly values (2003-2011) averaged per quarter[22]	1	https://hermes.acri.fr/

Table S2 | Country-level summary statistics for the tidal marsh global soil organic carbon (SOC) map. For each soil layer (0-30 cm and 30-100 cm), the initial predicted SOC stock, the proportion of the realm within the area of applicability (AOA), and the final predicted SOC stock, after masking out areas outside the AOA. The expected model error is shown in parentheses for each prediction. Only countries with a tidal marsh extent greater than 10 km^2 are represented here.

	0-30 cm			30-100 cm				
**Country**	**Initial predicted SOC stock (Mg ha^{-1})**	**Locations within the area of applicability (%)**	**Final predicted SOC stock (Mg ha^{-1})**	**Initial predicted SOC stock (Mg ha^{-1})**	**Locations within the area of applicability (%)**	**Final predicted SOC stock (Mg ha^{-1})**	**Area (km^2)**	**Total C to 1 m (Tg)**
Albania	80.38 (50.14)	94.8	79.79 (49.87)	169.69 (119.07)	82.2	162.90 (117.60)	79.01	1.92 (1.32)
Algeria	69.70 (51.29)	78.5	68.98 (50.1)	148.01 (121.23)	67.4	142.25 (117.78)	26.92	0.57 (0.45)
Angola	71.06 (53.22)	68.5	69.49 (52.75)	145.73 (125.95)	0	141.75 (123.97)	17.04	0.36 (0.30)
Argentina	78.84 (53.28)	43.4	77.46 (51.73)	166.46 (126.78)	9.2	166.75 (121.04)	2429.48	59.33 (41.97)
Australia	86.12 (47.41)	82.8	88.08 (45.69)	170.04 (116.91)	64.7	172.86 (110.87)	2080.51	54.29 (32.57)
Bahamas	73.89 (51.56)	80.7	74.24 (51.16)	156.85 (123.63)	39.3	154.76 (120.45)	116.29	2.66 (2.00)
Bangladesh	54.59 (58.04)	9.3	56.80 (52.81)	118.82 (137.07)	0.7	127.67 (122.04)	202.47	3.74 (3.54)
Belize	77.53 (49.18)	81.1	76.67 (47.71)	163.39 (118.6)	73.7	159.31 (115.09)	64.17	1.51 (1.04)
Benin	78.45 (52.83)	70.9	78.53 (52.33)	165.67 (124.67)	5.7	168.67 (122.27)	53.94	1.33 (0.94)
Brazil	73.61 (52.52)	58.3	76.11 (49.4)	164.26 (126.19)	34.7	171.06 (117.32)	1066.11	26.35 (17.77)
Bulgaria	96.09 (52.15)	93.2	95.48 (52.01)	200.18 (122.97)	58.5	193.87 (121.78)	19.55	0.57 (0.34)
Cambodia	79.76 (52.62)	76.7	80.04 (52.14)	165.66 (124.28)	34.4	163.94 (121.92)	15.8	0.39 (0.28)
Canada	94.11 (60.56)	8.5	104.97 (49.03)	186.27 (143.71)	3.2	227.79 (115.79)	8534.32	283.99 (140.66)
Chile	116.86 (54.01)	39.6	103.14 (49.97)	234.62 (127.9)	33.4	201.6 (118.49)	458.62	13.98 (7.73)
China	57.43 (50.88)	80.6	57.28 (49.97)	124.07 (121.81)	34.7	108.41 (117.65)	1167.51	19.34 (19.57)
Colombia	77.11 (53.58)	50	77.55 (52.03)	169.39 (126.63)	18.3	171.12 (121.27)	45.93	1.14 (0.80)
Costa Rica	71.7 (53.55)	38.4	71.98 (53.06)	148.61 (125.81)	2.6	149.71 (122.89)	15.00	0.33 (0.26)
Croatia	93.53 (52.30)	61.8	91.66 (51.24)	202.58 (124.31)	29.6	193.42 (119.34)	51.76	1.48 (0.88)
Cuba	85.10 (50.53)	97.4	85.22 (50.44)	178.55 (119.92)	90.9	179.13 (119.44)	783.79	20.72 (13.32)
Denmark	87.45 (48.54)	91.3	86.56 (47.93)	171.42 (118.18)	48.6	162.38 (113.05)	263.73	6.57 (4.25)
Dominican Republic	88.12 (51.91)	82.2	86.42 (51.4)	195.03 (123.26)	41.8	174.84 (120.22)	17.37	0.45 (0.30)
Egypt	71.26 (49.40)	94.8	70.60 (49.12)	146.63 (117.09)	92.3	144.07 (116.26)	176.83	3.80 (2.92)
Estonia	114.09 (45.82)	93.1	113.74 (45.09)	219.54 (116.9)	23.4	211.06 (113.97)	269.45	8.75 (4.29)
France	78.41 (49.83)	89.8	77.83 (49.23)	162.98 (118.64)	81.6	158.25 (116.74)	678.47	16.02 (11.26)
Gabon	77.45 (53.28)	65.6	76.30 (52.27)	162.03 (125.04)	53.6	159.02 (122.01)	34.69	0.82 (0.60)
Gambia	63.29 (53.95)	24.3	61.6 (53.00)	132 (127.25)	0.3	145.79 (122.13)	40.5	0.84 (0.71)

Georgia	89.32 (51.39)	86.1	88.01 (50.93)	234.36 (121.84)	63.2	232.56 (119.57)	65.04	2.08 (1.11)
Germany	78.36 (46.39)	97.6	77.71 (46.19)	157.88 (113.02)	71.9	143.10 (109.33)	346.03	7.64 (5.38)
Ghana	71.94 (52.63)	90.1	70.95 (52.46)	145.83 (124.38)	5.8	146.91 (122.5)	16.96	0.37 (0.30)
Greece	81.27 (49.85)	93.6	80.75 (49.57)	170.87 (118.55)	83.2	166.81 (117.18)	213.35	5.28 (3.56)
Guatemala	90.00 (53.03)	62.1	92.11 (51.98)	192.07 (125.12)	33.8	199.25 (121.86)	11.56	0.34 (0.20)
Guinea-Bissau	63.46 (57.03)	10.0	59.19 (52.23)	136.49 (134.55)	2.2	125.01 (121.68)	68.82	1.27 (1.2)
Guyana	74.00 (59.96)	0	NA	170.37 (141.28)	0	NA	33.28	NA
Haiti	73.48 (53.70)	42.8	72.45 (52.39)	157.60 (127.00)	4.1	184.12 (119.60)	38.64	0.99 (0.66)
Honduras	81.24 (54.44)	18.7	74.83 (52.92)	175.59 (128.01)	0.3	150.88 (121.36)	393.12	8.87 (6.85)
India	64.84 (55.75)	31.1	69.72 (52.13)	139.07 (132.23)	8.1	153.00 (120.2)	52.3	1.16 (0.9)
Indonesia	75.82 (59.06)	0.4	86.01 (52.32)	178.56 (139.57)	0.1	180.18 (122.09)	170.73	4.54 (2.98)
Iran	92.16 (54.00)	16.4	64.41 (46.96)	209.04 (127.41)	15.5	128.88 (114.06)	11.10	0.21 (0.18)
Ireland	88.31 (45.36)	96.4	87.56 (45.06)	165.85 (108.88)	90.4	162.14 (106.94)	130.69	3.26 (1.99)
Italy	78.54 (48.43)	97.0	77.40 (48.24)	164.25 (115.97)	89.3	160.04 (114.87)	228.72	5.43 (3.73)
Jamaica	83.31 (51.69)	97.4	83.21 (51.64)	176.45 (122.59)	51.5	176.65 (121.61)	20.68	0.54 (0.36)
Japan	86.14 (54.01)	43.0	84.16 (51.62)	180.90 (128.61)	4.2	212.61 (119.15)	267.64	7.94 (4.57)
Latvia	95.12 (49.23)	89.5	94.87 (48.44)	184.26 (120.88)	25.0	177.95 (117.04)	163.61	4.46 (2.71)
Lithuania	87.18 (49.67)	85.7	86.73 (48.77)	170.07 (121.39)	8.4	168.26 (117.76)	37.97	0.97 (0.63)
Madagascar	71.52 (53.96)	37.6	65.06 (51.27)	152.57 (127.15)	26.2	131.66 (119.89)	153.32	3.02 (2.62)
Mauritania	70.38 (50.08)	98.8	70.45 (50.03)	139.34 (119.85)	68.0	144.5 (118.18)	28.26	0.61 (0.48)
Mexico	79.68 (49.59)	90.7	79.25 (49.06)	171.64 (118.79)	79.6	169.13 (116.82)	1194.69	29.67 (19.82)
Montenegro	90.85 (54.28)	31.4	92.88 (50.81)	194.46 (129.49)	18.1	192.47 (118.95)	26.66	0.76 (0.45)
Morocco	59.22 (48.77)	87.5	58.37 (48.05)	114.95 (116.53)	75	108.94 (113.35)	43.02	0.72 (0.69)
Mozambique	63.81 (52.96)	55.8	63.72 (51.70)	135.42 (125.15)	29.5	131.52 (121.00)	1343.19	26.22 (23.20)
Myanmar	59.41 (68.44)	0	NA	129.19 (162.63)	0	NA	122.99	NA
Netherlands	82.41 (47.24)	86.5	75 (45.8)	181.83 (114.78)	79.1	160.04 (110.98)	183.33	4.31 (2.87)
New Zealand	92.06 (52.51)	70.0	90.14 (49.46)	183.97 (125.09)	53.9	175.11 (116.50)	244.82	6.49 (4.06)
Nicaragua	76.51 (54.95)	12.9	79.17 (52.99)	162.46 (128.80)	8.5	162.68 (123.64)	559.72	13.54 (9.89)
Nigeria	78.18 (54.01)	31.7	79.77 (52.82)	169.71 (127.22)	3.8	157.82 (123.56)	21.42	0.51 (0.38)
North Korea	63.56 (53.57)	41.4	58.94 (52.26)	132.06 (125.35)	35.6	122.07 (121.92)	80.01	1.45 (1.39)
Papua New Guinea	81.76 (58.32)	3.0	88.16 (52.82)	185.78 (138.09)	0.2	186.95 (122.52)	53.25	1.46 (0.93)
Peru	72.84 (52.37)	68.9	71.28 (51.43)	145.87 (125.54)	13.0	129.47 (120.02)	10.08	0.20 (0.17)
Poland	95.84 (49.29)	95.3	95.28 (49.03)	183.46 (120.51)	20.0	179.49 (118.04)	161.17	4.43 (2.69)
Portugal	58.56	97.6	58.33	109.84	96.3	108.87	157.7	2.64

	(44.17)		(43.89)	(108.51)		(107.71)		(2.39)
Romania	91.26 (51.93)	97.3	91.21 (51.88)	187.77 (123.24)	28.7	187.15 (122.28)	667.23	18.57 (11.62)
Russia	106.10 (56.11)	26.2	91.10 (51.04)	208.2 (132.76)	20.8	180.12 (119.29)	5140.54	139.42 (87.56)
Senegal	63.90 (51.45)	95.5	63.93 (51.33)	127.10 (122.15)	37.0	126.67 (120.94)	52.43	1.00 (0.9)
Sierra Leone	67.96 (57.92)	0	NA	141.94 (136.25)	0	NA	21.08	NA
South Africa	82.69 (47.23)	96.6	82.32 (46.97)	165.13 (114.6)	81.0	158.85 (112.5)	89.15	2.15 (1.42)
South Korea	61.36 (54.42)	18.3	63.12 (52.85)	125.03 (127.32)	6.6	114.86 (123.52)	180.83	3.22 (3.19)
Spain	62.07 (46.21)	98.3	61.99 (46.05)	117.78 (111.96)	95.8	116.61 (111.3)	341.47	6.10 (5.37)
Suriname	69.21 (57.06)	0	NA	157.88 (134.34)	0	NA	42.44	NA
Sweden	94.47 (49.54)	87.9	93.3 (48.69)	178.61 (120.88)	31.5	164.59 (115.81)	117.08	3.02 (1.93)
Thailand	82.76 (54.15)	40	86.4 (51.92)	180.60 (127.74)	21	185.52 (121.33)	18.25	0.50 (0.32)
Tunisia	66.59 (46.31)	98.5	65.53 (46.15)	134.90 (109.91)	97.8	131.81 (109.43)	53.80	1.06 (0.84)
Turkey	88.35 (49.08)	94	88.35 (48.75)	189.20 (117.65)	82.9	187.61 (116)	252.44	6.97 (4.16)
Ukraine	87.80 (52.14)	87.4	87.64 (51.76)	175.85 (123.52)	38.7	172.70 (121.95)	923.82	24.05 (16.05)
United Kingdom	88.53 (42.84)	98	87.97 (42.6)	171.60 (105.58)	93.2	167.37 (104.12)	535.86	13.68 (7.86)
United States	86.71 (42.45)	84.4	82.76 (39.99)	201.63 (104.27)	82	198.86 (98.54)	18509.72	521.27 (256.42)
Uruguay	83.39 (51.17)	85.1	83.02 (50.5)	185.71 (122.46)	50.6	183.21 (119.55)	286.62	7.63 (4.87)
Venezuela	79.24 (54.59)	30.1	80.62 (51.32)	174.25 (129.38)	13.1	178.49 (119.04)	165.20	4.28 (2.81)
Vietnam	77.34 (54.97)	36.8	81.24 (52.51)	165.93 (129.91)	13.4	166.02 (122.72)	12.08	0.30 (0.21)

Table S3 | Realm level summary statistics for the tidal marsh global soil organic carbon (SOC) map. For each soil layer (0-30 cm and 30-100 cm), we present the initial predicted SOC stock, the proportion of the realm within the area of applicability (AOA), i.e. where we enabled the model to learn about the relationship between SOC stocks and the environmental drivers, and the final predicted SOC stock, after masking out areas outside the AOA. The estimated model error is shown in parentheses for each prediction. Realms correspond to the biogeographical realms of the Marine Ecoregions of the World[1].

	**0-30 cm**			**30-100 cm**		
**Realm**	**Initial predicted SOC stock (Mg ha^{-1})**	**Pixels within the area of applicability (%)**	**Final predicted SOC stock (Mg ha^{-1})**	**Initial predicted SOC stock (Mg ha^{-1})**	**Pixels within the area of applicability (%)**	**Final predicted SOC stock (Mg ha^{-1})**
Arctic	96.19 (60.55)	1.5	121.80 (52.49)	187.67 (143.15)	0.3	252.54 (122.05)
Central Indo-Pacific	70.55 (56.3)	22.6	71.35 (51.09)	155.06 (133.03)	13.4	149.19 (118.86)
Eastern Indo-Pacific	82.06 (54.45)	31.3	83.88 (52.44)	167.15 (128.98)	5.0	188.53 (120.9)
Temperate Australasia	89.56 (47.10)	87.9	89 (45.87)	176.2 (116.30)	68.9	174.04 (111.19)
Temperate Northern Atlantic	84.95 (43.56)	95.2	84.24 (42.98)	192.62 (106.58)	81.8	190.87 (102.60)
Temperate Northern Pacific	98.34 (54.80)	29.2	77.11 (48.19)	196.24 (130.45)	17.7	161.19 (112.22)
Temperate South America	84.41 (52.75)	52.0	81.16 (50.75)	178.87 (125.75)	22.4	180.69 (118.75)
Temperate Southern Africa	83.31 (47.13)	96.4	82.96 (46.84)	165.96 (114.48)	78.8	159.59 (112.08)
Tropical Atlantic	77.52 (51.65)	65.4	78.77 (49.46)	166.04 (123.30)	53.4	168.92 (117.76)
Tropical Eastern Pacific	82.72 (50.44)	67.7	86.42 (48.5)	178.76 (120.81)	45.6	195.89 (113.88)
Western Indo-Pacific	63.56 (54.63)	44.9	63.95 (51.58)	135.55 (129.16)	24.1	131.97 (120.60)
Global	88.13 (50.63)	58.0	83.09 (44.77)	186.60 (121.69)	46.2	185.27 (105.71)

Supplementary References

1. Spalding, M. D. *et al.* Marine ecoregions of the world: A bioregionalization of coastal and shelf areas. *Bioscience* **57**, 573–583 (2007).
2. Linnenbrink, J., Milà, C., Ludwig, M. & Meyer, H. kNNDM: k-fold Nearest Neighbour Distance Matching Cross-Validation for map accuracy estimation. *EGUsphere* https://doi.org/10.5194/egusphere-2023-1308 (2023) doi:10.5194/egusphere-2023-1308.
3. Young, M. A. *et al.* National scale predictions of contemporary and future blue carbon storage. *Sci. Total Environ.* **800**, 149573 (2021).
4. Ewers Lewis, C. J. *et al.* Drivers and modelling of blue carbon stock variability in sediments of southeastern Australia. *Biogeosciences* **17**, 2041–2059 (2020).
5. Duarte de Paula Costa, M. *et al.* Current and future carbon stocks in coastal wetlands within the Great Barrier Reef catchments. *Glob. Chang. Biol.* **27**, 3257–3271 (2021).
6. Martinetto, P. *et al.* The blue carbon of southern southwest Atlantic salt marshes and their biotic and abiotic drivers. *Nat. Commun.* **14**, 1–10 (2023).
7. Murray, N. J. *et al.* High-resolution mapping of losses and gains of Earth’s tidal wetlands. *Science (80-.).* **376**, 744–749 (2022).
8. Murray, N. nick-murray/gee-global-intertidal-change-ARCHIVE: v1.0 for manuscript publication. at https://doi.org/10.5281/zenodo.6503069 (2022).
9. Connor, R. F., Chmura, G. L. & Beecher, C. B. Carbon accumulation in Bay of Fundy salt marshes: Implications for restoration of reclaimed marshes. *Global Biogeochem. Cycles* **15**, 943–954 (2001).
10. Rogers, K., Macreadie, P. I., Kelleway, J. J. & Saintilan, N. Blue carbon in coastal landscapes: A spatial framework for assessment of stocks and additionality. *Sustain. Sci.* **14**, 453–467 (2019).
11. Rogers, K. *et al.* Wetland carbon storage controlled by millennial-scale variation in relative sea-level rise. *Nature* **567**, 91–95 (2019).

12. Hayes, M. A. *et al.* Dynamics of sediment carbon stocks across intertidal wetland habitats of Moreton Bay, Australia. *Glob. Chang. Biol.* **23**, 4222–4234 (2017).
13. Spivak, A. C., Sanderman, J., Bowen, J. L., Canuel, E. A. & Hopkinson, C. S. Global-change controls on soil-carbon accumulation and loss in coastal vegetated ecosystems. *Nat. Geosci.* **12**, 685–692 (2019).
14. Rogers, K. Accommodation space as a framework for assessing the response of mangroves to relative sea-level rise. *Singap. J. Trop. Geogr.* **42**, 163–183 (2021).
15. Clark, J. A., Farrell, W. E. & Peltier, W. R. Global changes in postglacial sea level: A numerical calculation. *Quat. Res.* **9**, 265–287 (1978).
16. Rovai, A. S. *et al.* Global controls on carbon storage in mangrove soils. *Nat. Clim. Chang.* **8**, 534–538 (2018).
17. Smith, A. J., Noyce, G. L., Megonigal, J. P., Guntenspergen, G. R. & Kirwan, M. L. Temperature optimum for marsh resilience and carbon accumulation revealed in a whole-ecosystem warming experiment. *Glob. Chang. Biol.* **28**, 3236–3245 (2022).
18. Serrano, O. *et al.* Australian vegetated coastal ecosystems as global hotspots for climate change mitigation. *Nat. Commun.* **10**, 1–10 (2019).
19. Easterling, D. R. *et al.* Climate extremes: Observations, modeling, and impacts. *Science (80-.).* **289**, 2068–2074 (2000).
20. Hughes, C. E., Kalma, J. D., Binning, P., Willgoose, G. R. & Vertzonis, M. Estimating evapotranspiration for a temperate salt marsh, Newcastle, Australia. *Hydrol. Process.* **15**, 957–975 (2001).
21. Dethier, E. N., Renshaw, C. E. & Magilligan, F. J. Rapid changes to global river suspended sediment flux by humans. *Science (80-.).* **376**, 1447–1452 (2022).
22. Sanderman, J. *et al.* A global map of mangrove forest soil carbon at 30 m spatial resolution. *Environ. Res. Lett.* **13**, 055002 (2018).
